# Novel Insights into Pituitary Tumorigenesis: Genetic and Epigenetic Mechanisms

**DOI:** 10.1210/endrev/bnaa006

**Published:** 2020-03-23

**Authors:** Vinaya Srirangam Nadhamuni, Márta Korbonits

**Affiliations:** Centre for Endocrinology, William Harvey Research Institute, Barts and the London School of Medicine and Dentistry, Queen Mary University of London, UK

**Keywords:** pituitary neoplasm, pituitary tumorigenesis, pituitary adenoma, PitNET, pituitary tumor

## Abstract

Substantial advances have been made recently in the pathobiology of pituitary tumors. Similar to many other endocrine tumors, over the last few years we have recognized the role of germline and somatic mutations in a number of syndromic or nonsyndromic conditions with pituitary tumor predisposition. These include the identification of novel germline variants in patients with familial or simplex pituitary tumors and establishment of novel somatic variants identified through next generation sequencing. Advanced techniques have allowed the exploration of epigenetic mechanisms mediated through DNA methylation, histone modifications and noncoding RNAs, such as microRNA, long noncoding RNAs and circular RNAs. These mechanisms can influence tumor formation, growth, and invasion. While genetic and epigenetic mechanisms often disrupt similar pathways, such as cell cycle regulation, in pituitary tumors there is little overlap between genes altered by germline, somatic, and epigenetic mechanisms. The interplay between these complex mechanisms driving tumorigenesis are best studied in the emerging multiomics studies. Here, we summarize insights from the recent developments in the regulation of pituitary tumorigenesis.

## Graphical Abstract

**Graphical Abstract:**
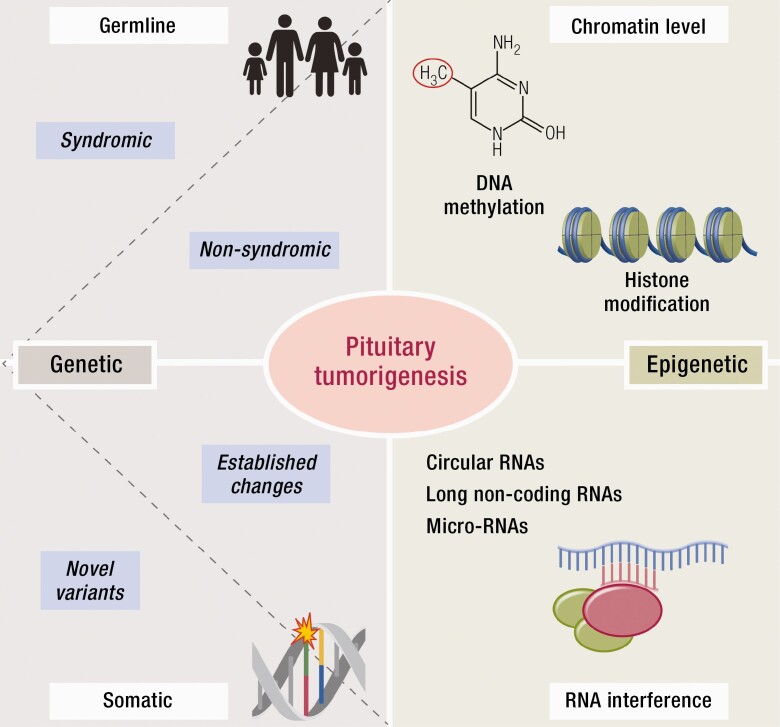

**Essential Points**

- An increasing number of genes with germline mutations are known now to be associated with pituitary tumors, some causing syndromic disease while others isolated pituitary adenomas.
- Gain-of-function somatic mutations are common in somatotropinomas in the *GNAS* gene and in corticotropinomas in *USP8*.
- Other, less common somatic variants recently identified through next generation sequencing need to be confirmed in independent cohorts and elucidated through functional studies in the future.
- Epigenetic modifications (DNA methylation, histone modification, and noncoding RNAs) can greatly influence tumorigenesis and tumor characteristics such as subtype differentiation and local invasion.
- An integrated multiomics approach to characterize genetic and epigenetic pathways allows better understanding the molecular mechanisms that underlie pituitary tumorigenesis within and across the various subtypes and may lead to the identification of better prognostic factors.

Pituitary tumors (PTs) are common intracranial neoplasms with an overall prevalence estimated at 17% in a systematic review using post-mortem (14%) and radiologic studies (22%) (1). While the majority of these would represent incidentalomas and usually of little clinical significance, the prevalence of clinically-presenting adenomas is higher in epidemiological studies conducted over the last 10–15 years compared to older data, probably due to better diagnostic modalities, with 68–110 PTs clinically-presenting cases identified per 100 000 inhabitants (2–8). Using incidence data from population-based state cancer registries in the United States, the age-adjusted annual incidence rate of PTs increases from 2.52 in 2004 to 3.13 in 2009 (per 100 000 subjects) (9).

Tumors of the anterior pituitary usually do not metastasize, and hence have been referred to as “adenomas.” However, as a significant minority can show clinically aggressive behavior and similar characteristics to true metastasizing lesions (10), the term “pituitary neuroendocrine tumor” or “PitNET” has been coined recently, receiving mixed acceptance (11–14). Here we use the term pituitary tumor representing tumors arising from the potentially hormone-producing cells of the anterior pituitary.

Pituitary tumors are clinically categorized by their hormone-secreting characteristics, with over-secretion of growth hormone (GH), prolactin, adrenocorticotropic hormone (ACTH), thyroid stimulating hormone (TSH), luteinizing hormone (LH) and follicle-stimulating hormone (FSH) or clinically nonfunctioning tumors. Histological characterization has been based on immunohistochemical staining of pituitary hormones, with more recently transcription factors (PIT1 for GH, prolactin and TSH lineages, SF1 for gonadotroph lineages and TPIT for ACTH lineage) being added to the classification (15). Therefore, the final diagnosis relies on the combination of the clinical picture (excess hormone secreting or not) and histological assessment (hormone and transcription factor immunostaining (16)). Molecular characterization based on methylation patterns, gene expression and DNA mutations may add further granularity to the assessment of these tumors in the future (17).

Over the last decade, we have witnessed major advances in the biology of pituitary tumors, with the identification of several germline and somatic mutations and epigenetic mechanisms, such as DNA methylation, histone modifications, and noncoding RNAs. In this review, genetic and epigenetic mechanisms contributing to pituitary tumorigenesis will be succinctly summarized with an emphasis on novel insights over the last ten years.

## Genetic Mechanisms Of Tumorigenesis

### Germline mutations driving tumorigenesis

**Figure 1.**
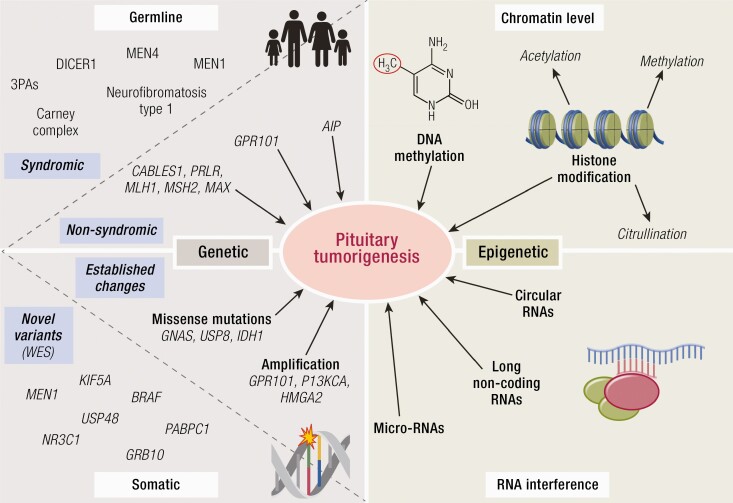
Genetic and epigenetic mechanisms of pituitary tumorigenesis. Genetic mechanisms may be secondary to germline or somatic mutations, while epigenetic mechanisms can be mediated at the chromatin level (such as in the case of DNA methylation or histone modifications) or via noncoding RNAs.

**Figure 2.**
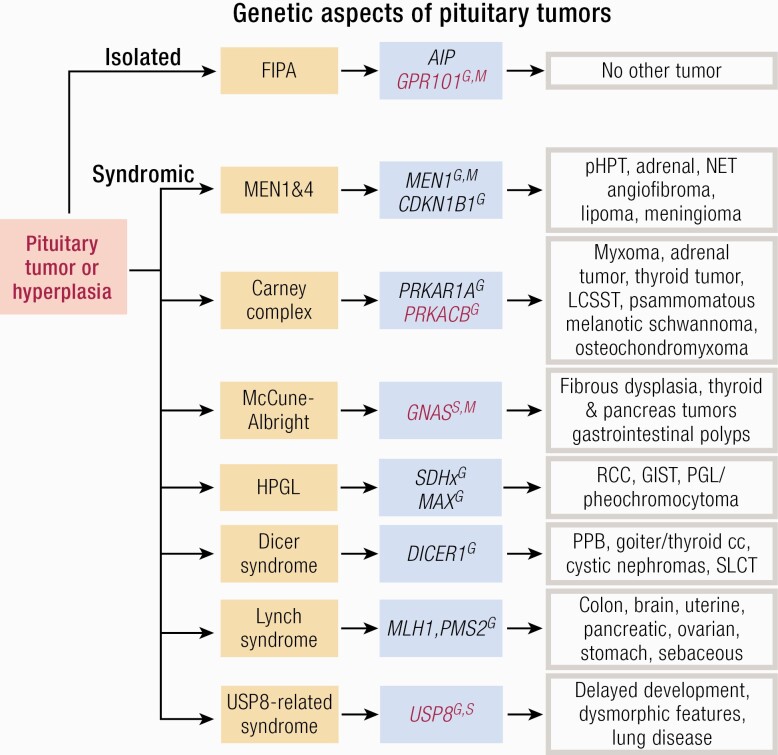
Germline or mosaic mutations causing pituitary tumors. Pituitary tumors presenting in isolation (familial isolated pituitary adenoma, FIPA) or part of a tumor syndrome. Hyperplasia has been described in Carney complex, McCune-Albright syndrome, 20% of XLAG cases and rarely in *AIP* mutation positive cases. Genes marked with red letter types are oncogenes, while the black ones are tumor suppressor genes. Abbreviations: G, germline; GIST, gastrointestinal stromal tumor; HPGL, hereditary paraganglioma; LCCSCT, large-cell calcifying Sertoli cell tumors; M, mosaic; NET, neuroendocrine tumor; pHPT, primary hyperparathyroidism; PPB, pleuropulmonary blastoma; RCC, renal cell carcinoma; S, somatic; SLCT, Sertoli–Leydig cell tumor.

Pituitary tumors associated with germline mutations may present as part of a syndromic disease or in isolation (Fig. 1, 2). The nonsyndromic group consists of patients in whom no other organ than the pituitary is involved, and is known as familial isolated pituitary adenoma (FIPA) (18). We summarize here the key genetic aspects, while refer to other reviews on the detailed clinical characteristics of these diseases (19, 20).

#### Familial isolated pituitary adenoma (FIPA).

The prevalence of FIPA among all PT patients was found to be 1.9% to 3.8% in pituitary referral centers (2, 21). The first identified gene underlying FIPA is *AIP* (22), which accounts for 10% to 20% of FIPA kindreds (23, 24). Duplication of *GPR101* in X-linked acrogigantism (XLAG), although mostly identified as *de novo* mutation, has also been described in families (three kindreds described so far in the literature (25–28)). However, patients with a suggestive family history with no known genetic cause form the majority of patients with FIPA.

##### AIP mutation-positive pituitary tumors.

**Figure 3.**
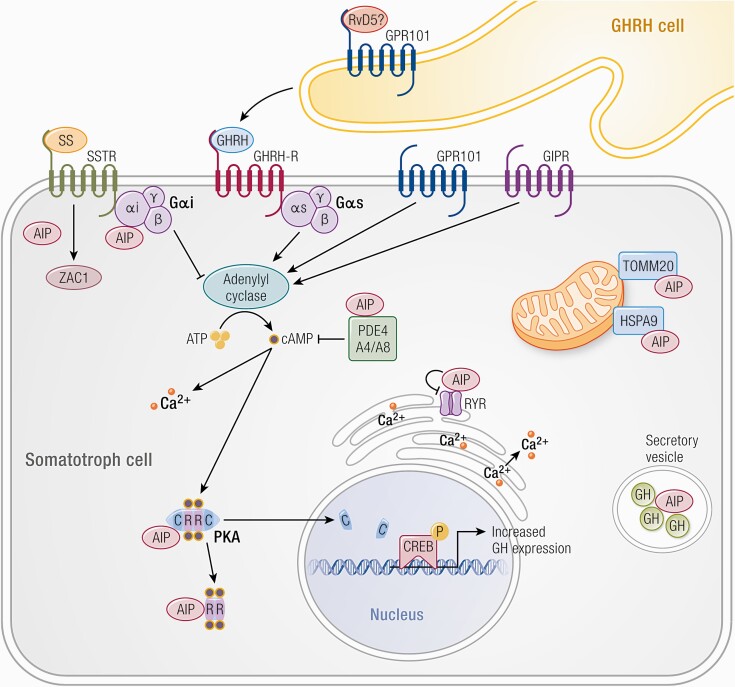
Tumorigenic mechanism in somatotroph cells. cAMP-associated pathways are key for somatotroph tumorigenesis. GHRH released by the hypothalamus interacts with its receptor (GHRH-R) on the somatotroph cell membrane to increase activation of adenylyl cyclase through Gαs. Consequent increased cAMP production leads to the dissociation of the regulatory subunits (R) of protein kinase A (PKA) from the catalytic subunits (C), which then translocate to the nucleus and phosphorylate CREB (cAMP response element) and other targets, eventually leading to increased GH expression and cell proliferation. Mosaic (McCune-Albright syndrome) or somatic activating mutations in *GNAS* (coding for Gαs) lead to upregulation of the cAMP pathway. In Carney complex, increased PKA activity, either due to the inhibitory action of the regulatory subunit PRKAR1A, or increased catalytic subunit activity (PRKACB) leads to tumorigenesis. Loss of AIP has been shown to increase cAMP signaling through (1) decreased expression of the G inhibitory protein Gαi-2, which mediates the inhibitory effects of somatostatin (SS) on adenylyl cyclase. AIP deficiency is associated with reduced Gαi-2 expression in human and mouse GH-PTs (2, 32) an interaction with phosphodiesterases type 4 (PDE4) (36); expression of type 4 phosphodiesterase is lower in *AIP*-mutated GH-PTs compared to sporadic GH-PTs (37) and *AIP* mutations disrupt the interaction of AIP with PDE4A5 in GH3 cell (3, 35) interaction of AIP with members of the PKA complex (38, 39). AIP deficiency results in reduced ZAC1 levels (40, 41) and is associated with mitochondrial proteins TOMM20 and HSPA9 (39, 69), the endoplasmatic reticulum calcium channel RYR (31) and with secretory vesicles (35), but the exact mechanisms as to how these interactions might lead to tumorigenesis are unclear. GPR101 is Gsα-coupled constitutively active receptor leading to increased cAMP signaling. The mechanism of *GPR101*-related tumorigenesis may occur via a dual mechanism: hypothalamic dysregulation as elevated GHRH levels can be measured in some patients, while there may be a direct pituitary action due to increased GPR101 expression on pituitary cells. Recently an endogenous ligand has been identified, the lipid mediator Resolvin D5 (RvD5), the role of this mediator in the regulation of the GH axis and its levels in patients with XLAG is currently unknown. Ectopic expression of GIPR may also lead to an activated cAMP pathway (70–72).

The *AIP* gene maps to chromosome 11q13.2, incidentally close to the locus of the *MEN1* gene, although there are no sequence similarities between the two genes. It encodes a ubiquitously expressed co-chaperone protein with multiple partners, but currently its role in pituitary tumorigenesis is incompletely understood. It behaves as a tumor suppressor with a unique primarily somatotroph/lactotroph specificity, although global lack of *AIP* is lethal in mouse, *Drosophila* and *C. elegans* studies (29–31). The cAMP/protein kinase A/phosphodiesterase pathway plays a key role in somatotroph physiology and acromegaly-related genetic syndromes (Fig. 3). Not surprisingly, therefore, a link has been found between this pathway and AIP at several levels: at the inhibitory Gαi-2 protein (32, 33), at cAMP (34), at phosphodiesterase 4A (35-37), at protein kinase A (38, 39), downstream of somatostatin receptors, and Zac1 (40, 41) levels. AIP has also found interact and inhibit the endoplasmatic reticulum calcium channel ryanodine receptor in *C. elegans* (31), another pathway closely linked with hormone release, with somatic variants identified in calcium-related pathways in somatotropinomas (42, 43). Given the particular role of RET in somatotroph cells (44), the link with RET (45) could be a link to the specific role of AIP in somatotrophs. Increased GH release has been found in AIP-disrupted cells, probably associated with the increased STAT3 phosphorylation (41, 46), while an altered microenvironment may also explain the aggressive phenotype of some of these tumors (47). However, the role of other AIP partners—nuclear receptors (AHR, ER, GR, PPRα, TRβ1), mitochondrial proteins, survivin (reviewed in (48)) or BCL6 (49) in the pituitary-specific effects is unclear.

Pituitary tumors are significantly more frequent in global heterozygous *Aip*^*+/-*^ mice compared to wild-type mice (50). The majority of the tumors developed were GH-PTs, which were negative for AIP immunostaining. This predisposition to developing GH-PTs in the heterozygous state with loss of expression of AIP in the tumor is similar to the human clinical phenotype. Interestingly, full penetrance is achieved in global heterozygous *Aip*^*+/-*^ mice by 15 months in contrast to the 23% penetrance observed in a representative, large, thoroughly screened human family (51). The discrepancy between the mouse and human phenotype is likely to be due to genetic variability in humans. Supporting this hypothesis is that fact that, although using the same mouse line, phenotypic variability has been noted by another laboratory in the penetrance of pituitary tumors in global heterozygous *Aip*^*+/-*^ model with no pituitary tumors detected at 12 months of age (52), in contrast to Raitila et al where pituitary tumor incidence was greater than 80% by 12 months. Homozygous somatotroph-specific knockout showed over 80% penetrance of GH-PTs by 10 months (53), with animals showing features similar to acromegaly (increased body size and elevated serum GH and insulin-like growth factor 1). High penetrance has also been observed in pituitary-specific *Aip*-knockout both in heterozygote and homozygote cases by 12–15 months (47, 54).

Patients with germline *AIP* mutations have a clinical phenotype that is distinct from sporadic tumors: they show earlier disease onset and are diagnosed at a younger age, are larger, and are predominantly sparsely-granulated GH-secreting tumors, locally invasive and develop apoplexy (23, 24, 35, 55–57). These tumors are less likely to respond to first-generation somatostatin analogues (SSA) (35, 55, 58), while some cases have shown responses to the second-generation SSA pasireotide (59).

Families with *AIP* mutations show an incomplete penetrance of around 15% to 30% (22, 24, 51, 55). This probably explains why 50% to 70% of the identified *AIP* mutation positive kindreds do not have a known family history (simplex cases) (24, 55), while *de novo* mutations (60) are exceedingly rare. Genetic screening can identify carrier family members, and clinical screening leads to a surprisingly high percentage of earlier recognition of clinically relevant disease (24).

The prevalence of *AIP* mutations in patients with sporadic pituitary tumors varies significantly depending on age of disease onset, family history and tumor type (61). It is highest among patients with gigantism, 29% to 41% (62, 63), 12% in sporadic patients with age at diagnosis less than 30 years (64), and 3.6% of unselected population of a pituitary referral center (65). However, other studies found no pathogenic or likely pathogenic mutations, for example in a cohort of 127 adult PT patients less than 40 years at diagnosis (66), or in a group of 50 of SSA-resistant adult patients with acromegaly (58). Current recommendations suggest screening for *AIP* mutations in patients with no syndromic features and any of the following criteria: (1) childhood onset PT, (2) familial PT, or (3) a macroadenoma at age 30 years or younger (18, 67, 68).

##### X-linked acrogigantism (XLAG).

XLAG is an extremely rare condition showing early-onset gigantism secondary to germline or somatic microduplication of the Xq26.3 chromosomal region containing the *GPR101* gene (25).

GPR101 is an orphan G-protein coupled receptor, with constitutive activity of the human protein. GPR101 is predicted to couple to the Gα stimulatory protein (73), which activates adenylyl cyclase and increases cAMP production. Indeed, culture of pituitary tissue of an XLAG patient showed increased GH and prolactin release (74), and heterologous in vitro overexpression of human GPR101 leads to increased cAMP signaling in HEK293 cells (73) and GH3 cells (25). GPR101 is expressed in the normal human hypothalamus and in the embryonic and pubertal pituitary, but less in prepubertal and adult pituitary, suggesting a role in development and at the peak of growth (75). GPR101 is overexpressed in XLAG pituitary tumors, while expression in sporadic GH-PTs is low (25). The hypothalamic expression could explain the increased GHRH levels measured in some of the patients, and points to the role of hypothalamic dysregulation in this disease (74).

Recently, a lipid mediator n-3 docosapentaenoic-derived resolvin D5 (7S,17S-dihydroxy-8E,10Z,13Z,15E,19Z-docosapentaenoic acid, RvD5n-3 DPA) has been shown to activate GPR101, representing a potential endogenous ligand to this previously orphan receptor (76). This bioactive lipid mediator enzymatically derived from essential fatty acid n-3 docosapentaenoic acid is a member of the specialized proresolving mediators. It plays a role in the regulation of leukocytes and macrophages, intestinal barrier protection, and in joint inflammation. It is currently unknown whether this ligand has a role in the physiological regulation of the GH axis and how it behaves in patients with XLAG. A previously suggested putative ligand for GPR101 is GnRH- (1–5), a short fragment of GnRH. GnRH- (1–5) has been suggested to activate GPR101 to increase epidermal growth factor release and increase MMP-9 enzymatic activity in endometrial cancer cell lines, facilitating cellular migration and leading to an increase in cellular invasion (77). Similar proproliferation and proinvasive effects may underlie pituitary tumorigenesis in XLAG.

The majority of the reported XLAG cases are sporadic (26 patients) due to de novo mutations, while three kindreds have been reported in the literature to date (25–28, 63, 74, 78–80). The majority of the patients are females (24/33, 73%), with all female cases showing de novo germline duplication, making transmission to future generations possible (81). In males, XLAG is secondary to somatic mosaicism in simplex cases described so far (78, 82), or to germline duplications inherited from an affected mother (full penetrance was seen in all three kindreds) (25–28). Two of 3 familial female patients described so far are mothers of affected sons and have de novo mutations; all 4 familial male patients are affected sons who have inherited the duplication from their mothers (25–28). It is unclear why de novo germline mutations have not been described in males so far.

Patients with XLAG have a distinct clinical phenotype with the onset of symptoms and diagnosis in early childhood (25, 63). Clinical presentation in most cases is due to accelerated growth velocity in infancy or early childhood (<5 years of age; most commonly during the first 2 years of life) (63), with acromegaly-type features such as acral enlargement and coarse facial features, signs which are often not seen in patients with other types of childhood-onset acromegaly (28). The majority (~80%) of the patients present with a GH and prolactin-secreting macroadenoma (25, 63). Some of the patients have a normal-sized pituitary gland or diffusely enlarged histologically-proven pituitary hyperplasia (63, 78, 83), despite very high levels of GH and IGF-1, with or without prolactin elevation. A prenatally diagnosed familial XLAG case showed a pituitary tumor on MRI already at 3 weeks of age, associated with high prolactin and growth hormone (26, 27). Given the full penetrance observed so far in familial XLAG, preimplantation diagnosis or prenatal screening is worth considering in affected mothers, and theoretically in female fetuses of affected males (although male-to-female transmission has not yet been demonstrated). Histological features show mixed GH-PRL tumors with a mixed sparsely and densely granulated pattern (27, 28, 63, 79). No other PT type has been associated with *GPR101* duplications (84). Missense variants of *GPR101* do not seem to be associated with PTs (63, 85, 86).

##### Novel germline variants.

Comprehensive reviews of clinical and genetic aspects of germline syndromes are available elsewhere: here we briefly summarize here the more recent developments (Fig. 1).

The vast majority of FIPA does not have an established genetic basis: approximately 85% of the FIPA cohort were negative for *AIP* mutations in a study (23). Consequently, there has been significant interest in identifying other germline variants, which may predispose to familial tumors (Table 1), but none of the published data convincingly supports the established presence of a further gene causing FIPA.

**Table 1.** Suggested germline variants, which may underlie FIPA or GH excess. Gene locations are according to the using human genome hg19/GRCh37 assembly.

Gene (Symbol)	Gene (Name)	Location	Association with Hormone-Secreting Subtype	Function of Gene Product and Mechanism of Tumorigenesis, if Known	In Vitro Evidence	In Vivo Evidence	Loss of Heterozygosity	Familial Presentation	References
CABLES1	Cdk5 and Abl enzyme substrate 1	18: 20,714,528-20,840,431	ACTH-PTs	Cell cycle progression: inhibits corticotroph cell proliferation.	Increased proliferation seen in corticotroph cells following knockdown using Cables1 small interfering RNA (88). All identified variants located close to the predicted cyclin-dependent kinase-3 (CDK3)-binding region of Cables1 and showed impaired ability to block cell proliferation in response to dexamethasone in corticotroph cells (87).	None available.	Not found for all variants.	Simplex for all identified variant.	(87)
PRLR	Prolactin receptor	5: 35,048,861-35,230,794	PRL-PTs	Prolactin receptor	Increased prolactin-induced AKT signaling and proliferation seen in p.Asn516Ile only (gain-of-function) (89). p.Ile100Val, p.Ile170Leu, p.Glu108Lys, and p.Glu554Gln have no effect on PRLR expression, localization, and signaling after prolactin stimulation in vitro, suggestive of minimal functional relevance (89, 90).	Female mice with a germline loss-of-function mutation in PRLR show large PRL-PTs (91) with a penetrance of 100% from 12 months of age (92).	Not investigated.	Gorvin et al: Familial in p.Ile100Val, simplex in p.Glu400Gln, p.Asp492Ile, unavailable for other variants (89). Bernard et al: simplex cases in all variants identified (p.Ile100Val, p.Ile170Leu, p.Glu108Lys and p.Glu554Gln) (90).	(89, 90)
RXRG	Retinoid X receptor gamma	1: 165,370,159-165,414,433	PRL-PTs	Forms dimers with ligands, increasing their DNA binding and transcriptional function. The identified variant p.R317H localizes to the ligand-binding domain of the protein and may disrupt interactions.	None available.	None available.	Not investigated..	Familial	(93)
TH	Tyrosine hydroxylase	11: 2,185,159-2,193,107	PRL-PTs	Converts L-tyrosine into L-3,4-dihydroxyphenylalanine (L-DOPA), the essential and rate-limiting step to formation of dopamine. Reduced dopaminergic activity leads to reduced inhibitory effects on lactotroph cells, increasing prolactin secretion.	primary cultures of human lactotroph tumor cells were transfected with an adenovirus vector containing a cDNA encoding a human tyrosine hydroxylase: transfection induced increased production of dopamine, resulting in the predicted biologic effect of decreased prolactin secretion (94).	adenovirus-mediated delivery of tyrosine hydroxylase reduces pituitary growth and circulating prolactin levels in a model of estrogen-induced pituitary tumors in rats (95).	Not investigated.	Familial.	(93)
CDH23	Cadherin related 23	10: 73,156,691-73,575,702	None specific	Calcium-dependent cell-cell adhesion glycoprotein	None available	None available.	Not investigated.	Familial .	(96)
IGSF1	Immunoglobulin superfamily member 1	X: 130,407,480-130,533,677	Somatomammotroph hyperplasia	Membrane glycoprotein with modified residue possibly altering interaction with an extracellular ligand.	Transfection of GH3 cells with the p.N604T IGSF1 variant did not significantly affect GH production compared to wild-type. The mutant protein showed the same pattern of maturation and stability as wild-type when expressed in heterologous cells and was detected in the plasma membrane (97).	Male Igsf1Δexon1 null mice show increased serum IGF1 at 10 weeks. Assessment of the knockout model (Igsf1Δ312) demonstrated enhanced pituitary *Gh* mRNA expression (98).	Not investigated.	Familial.	(99)

Four heterozygous germline missense variants were identified in *CABLES1* in four sporadic patients from a cohort of 182 patients with ACTH-PTs with functional evidence of loss of function for some of them (Table 1). No familial cases have been reported to date (87).

While loss of the *Prlr* leads to large pituitary tumors in mice, homozygous loss-of-function *PRLR* mutation in a human patient with hyperprolactinemia and agalactia had no pituitary tumor (100). On the contrary, a gain-of-function variant was identified in 9 out of 46 patients with PRL-PTs, representing a possible novel mechanism for prolactinoma tumorigenesis. In addition, 3 other rare and 2 low-frequency variants found in this cohort may represent benign changes (89). Further data are needed to confirm these findings. Furthermore, no loss or gain-of-function mutations could be identified in a cohort of young 88 patients with PRL-PTs (90) or in a cohort of 16 PRL-PT (17).

There are some further reports of germline variants in patients with pituitary tumors but without functional elucidation to define pathogenicity or mechanisms. Investigation into a family with isolated PRL-PTs (3 affected siblings) with whole exome sequencing showed novel, germline, potentially pathogenic variants in *RXRG* and *TH* (93), the latter of which may be relevant as it encodes tyrosine hydroxylase which mediates the rate-limiting step in the formation of dopamine which, in turn, negatively regulates prolactin secretion in the pituitary. Further cases or functional studies will strengthen this report.

Using whole exome sequencing, a study of 12 FIPA families identified four families with germline variants in *CDH23*, which were predicted to be pathogenic using *in silico* analysis. Tumors of these patients showed a reduced frequency of cavernous sinus invasion, compared to the rest of the familial patients. The identified variants were predicted to be loss-of-function changes and occurred in conserved motifs, suggestive for impaired protein function, although *CDH23* is a large gene and therefore is, in general, more likely to harbor sequence variants. Homozygous mutations in *CDH23* result in Usher syndrome, characterized by congenital sensorineural hearing loss, vestibular dysfunction and early-onset retinitis pigmentosa (101). Pituitary tumors have not been described in association with any of these problems (102). This study also describes 2 (out of 125) sporadic pituitary tumor patients with homozygous *CDH23* variants (96), but it is not specified whether these individuals showed clinical manifestations of Usher syndrome.

Interestingly, a recently-described syndrome, X-linked IGSF1 deficiency characterized by central hypothyroidism, macro-orchidism (103) and prolactin deficiency (104), can be associated with acromegaloid facial features, increased head circumference and increased total GH secretion and IGF-1 levels (98). Given that patients show hyperplasia rather than adenomas, this may be secondary to a failure of regulatory and feedback mechanisms. A germline variant in *IGSF1* was identified in three family members with gigantism (due to somatomammotroph hyperplasia, rather than adenoma) (97). No effect of the variant on protein expression, maturation, stability, or membrane trafficking was observed. The authors speculate that, given the prediction that the modified residue changes the surface charge in the 6^th^ immunoglobulin loop, this may alter IGSF1’s interaction with an extracellular partner, although this variant is reasonably common in the general population (minor allele frequency is 0.009, Table 2).

**Table 2.** Germline nonynonymous missense variants identified with predictions of pathogenicity using SIFT, PolyPhen, and Condel. Variants that could not be identified unambiguously have been excluded (PRLR, p.Arg477Trp (89), p.Glu108Lys (90), CDH23, p.Arg3138Trp, p.Arg2115His, p.Arg3138Trp, and p.Asp3296Asn (96)) and have not been included in the following table. The variant associated with TH is a truncating mutation and does not have any predictions of pathogenicity using the missense tools.

Gene (Symbol)	Location	HGVSc	HGVSp	gnomAD Allele Frequency	SIFT Interpretation	PolyPhen Interpretation	Condel Interpretation	References
CABLES1	18:20716258	ENST00000256925.7:c.532G>A	ENSP00000256925.7:p.Glu178Lys	0.0101	Deleterious low confidence	Benign	N/A	(87)
	18:20716444	ENST00000256925.7:c.718C>T	ENSP00000256925.7:p.Leu240Phe	0.000773	Deleterious low confidence	Probably damaging	Deleterious	
	18:20774429	ENST00000256925.7:c.935G>A	ENSP00000256925.7:p.Gly312Asp	0.0000601	Deleterious	Probably damaging	Deleterious	
	18:20817151	ENST00000256925.7:c.1388A>G	ENSP00000256925.7:p.Asp463Gly	not present	Deleterious	Probably damaging	Deleterious	
PRLR	5:35084704	ENST00000382002.5:c.241G>A	ENSP00000371432.5:p.Gly81Ser	0.0001328	Tolerated	Benign	Neutral	(89)
	5:35065862	ENST00000382002.5:c.1198G>C	ENSP00000371432.5:p.Glu400Gln	0.000898	Tolerated	Possibly damaging	Deleterious	
	5:35065513	ENST00000382002.5:c.1547A>T	ENSP00000371432.5:p.Asn516Ile	0.0008925	Deleterious	Possibly damaging	Deleterious	
	5:35084647	ENST00000382002.5:c.298A>G	ENSP00000371432.5:p.Ile100Val	0.04221	Tolerated	Benign	neutral	(89, 90)
	5:35072712	ENST00000382002.5:c.508A>C	ENSP00000371432.5:p.Ile170Leu	0.01884	Tolerated	Benign	neutral	
	5:35065328	ENST00000382002.5:c.1732G>C	ENSP00000371432.5:p.Glu578Gln	0.001195	Tolerated	Possibly damaging	Deleterious	(90)
RXRG	1:165378891	ENST00000359842.5:c.950G>A	ENSP00000352900.5:p.Arg317His	0.00002495	Deleterious	Probably damaging	Deleterious	(93)
TH	11:2186469	ENST00000381178.1:c.1420A>T	ENSP00000370571.1:p.Lys474Ter	not present	N/A	N/A	N/A	(93)
CDH23	10:73494028	ENST00000224721.6:c.4151G>T	ENSP00000224721.6:p.Arg1384Leu	not present	Deleterious	Probably damaging	Deleterious	(96)
IGSF1	X:130412680	ENST00000370903.3:c.1811A>C	ENSP00000359940.3:p.Asn604Thr	0.009383	Tolerated	Probably damaging	Deleterious	(97)

Abbreviations: HGVSc, Human Genome Variation Society (HGVS) coding sequence name; HGVSp, HGVS protein sequence name; SIFT, sorting intolerant from tolerant prediction tool.

#### Syndromes associated with pituitary tumors.

In addition to the previously well-described syndromes with multiple tumor types where pituitary tumors represent one of the possible manifestations, several novel syndromes have been described over the last few years (Fig. 2). The MEN1 syndrome is due to germline loss-of-function mutations of the *MEN1* gene. Ten percent of the cases could be *de novo* mutations, sometimes identified as mosaicism in the proband (105–107).

An MEN1-like clinical picture can be seen in MEN4 syndrome due to mutation in cyclin dependent kinase inhibitors, primarily p27 (*CDKN1B*) and rarely in p21 (*CDKN1A*), p15 (*CDKN2B*), and p18 (*CDKN2C*) (108, 109).

In Carney complex, in addition to loss-of-function mutations in the regulatory protein kinase A subunit *PRKAR1A*, gain-of-function has been described in the catalytic protein kinase A subunit *PRKACB* (110). The disease-causing gene associated to the 2p16 locus in Carney complex cases is unknown.

While pituitary tumors and pheochromocytoma are rarely seen in MEN1 syndrome, the constellation of paraganglioma, pheochromocytoma, and pituitary tumor (“3P” association) is now increasingly recognized in patients with *SDHx* mutations (111, 112) with a characteristic histological phenotype (111) and pituitary adenomas developing in a *Sdhb*-knockout mouse model (113). *MAX* mutations have been identified in 5 patients with pituitary tumor and pheochromocytoma (114–116). While loss-of-heterozygosity has not been shown yet, further data are needed to confirm a causal relationship between *MAX* mutations and pituitary tumors.

Corticotroph tumors have been recently identified in three tumor syndromes. DICER1 syndrome (loss-of-of-function *DICER1* mutations) has shown infantile-onset large pituitary blastoma in a few patients (117). Germline mutations in the mismatch repair pathway (*MLH1, PMS2, MSH2, MSH6)* lead to Lynch syndrome, an autosomal dominant inherited cancer syndrome associated with colorectal, endometrial, ovarian, and other carcinomas. Germline mutations in *MLH1* (118) and *MSH2* (119) have been identified in patients with aggressively growing ACTH-secreting tumors. While somatic variants were found in a single nonfunctioning tumor in 4 mismatch-related genes (120), microsatellite instability was not found in 107 sporadic pituitary tumor samples (121). While somatic USP8 mutations are commonly seen in corticotroph adenomas, a germline mutation has now also been described in a child with dysmorphic features, developmental delay and a corticotroph tumor (122), with a second similar case now under workup (Constantine Stratakis, NIH, personal communication).

Rarely, optic pathway gliomas cause high GH levels in neuroﬁbromatosis type 1 (*NF1*), while true pituitary adenomas are extremely rare. Pituitary tumors have been reported in patients with tuberous sclerosis (123–126). It is currently unclear if these are indeed related to the *TSC1* or *TSC2* mutations or are coincidental findings.

### Somatic mutations driving tumorigenesis

The most common recurrent somatic mutations occur in *GNAS* in somatotroph tumors, and in *USP8* in corticotroph tumors. Other somatic changes suggested to be associated with pituitary tumors include: *PIK3CA* amplification (127, 128), *IDH1* mutations (129, 130), *TP53* in pituitary carcinomas (131) and ACTH-PTs (132), and *HMGA2* amplification in PRL-PTs (133–135). *HRAS* mutations have been seen in pituitary carcinoma (136), while the report of complex 1 mitochondrial mutations in oncocytomas (137) await confirmation. A somatic frameshift mutation in the glucocorticoid receptor gene (*NR3C1*) resulting in premature termination of the coding sequence has been described in a patient with Nelson’s syndrome, which may contribute to tumor development by reducing glucocorticoid feedback on tumor cells (138). No coding region *NR3C1* mutation was found in 18 ACTH-PTs using Sanger sequencing (139), or in 18 *USP8* mutation-negative PTs using exome sequencing (132). A single patient with a de novo missense germline *NR3C1* mutation associated with an ACTH-PA has also been described (138), while a child with corticotroph adenoma and partial glucocorticoid resistance had no detectable *NR3C1* mutation (140). A recent study reported a novel somatic recurrent (“hotspot”) mutation in splicing factor 3 subunit B1 (p.R625H in *SF3B1*) in about 20% of prolactinomas in altogether 227 prolactinomas (141). This variant causes aberrant splicing of the estrogen related receptor gamma (ESRRG), causing stronger activation of PIT-1 leading to prolactin secretion and lactotroph proliferation. This variant was not reported in another study sequencing prolactinoma 16 tissues (17). Variants discovered recently through next generation sequencing-based approaches are also discussed below.

#### GNAS.

*GNAS* encodes the stimulatory α subunit of G-proteins and shows the most frequent somatic mutations in GH-PTs, more recently confirmed through whole genome (WGS) (42) and whole exome sequencing (WES) (17, 120, 142, 143). Mutations affect codon 201 or 227, disrupting the GTPase activity of the protein (144) and leading to prolonged adenylyl cyclase activity and increased cAMP levels, driving tumorigenesis (Fig. 3). *GNAS*-mutated tumors are smaller (145–149), less likely to be locally invasive (149), and more likely to respond to SSAs (149, 150), although a Brazilian cohort showed no differences in tumor extension or response to SSAs between mutated and nonmutated tumors (151). Dopamine receptor 2 expression is increased in *GNAS*-mutated tumors, potentially allowing for *GNAS* mutation status in predicting the response to dopamine agonists in GH-PTs (17). Recently, DNA methylation-activated inhibitory Gα (Gαi) -signaling was found in *GNAS*-mutation-positive GH-PTs (152). Patients with somatic mosaicism for codon 201 *GNAS* develop McCune-Albright syndrome, characterized by somato- or somatomammotroph hyperplasia or tumor, polyostotic fibrous dysplasia, cafe-au-lait spots, and precocious puberty (153).

#### USP8 and USP48.

**Figure 4.**
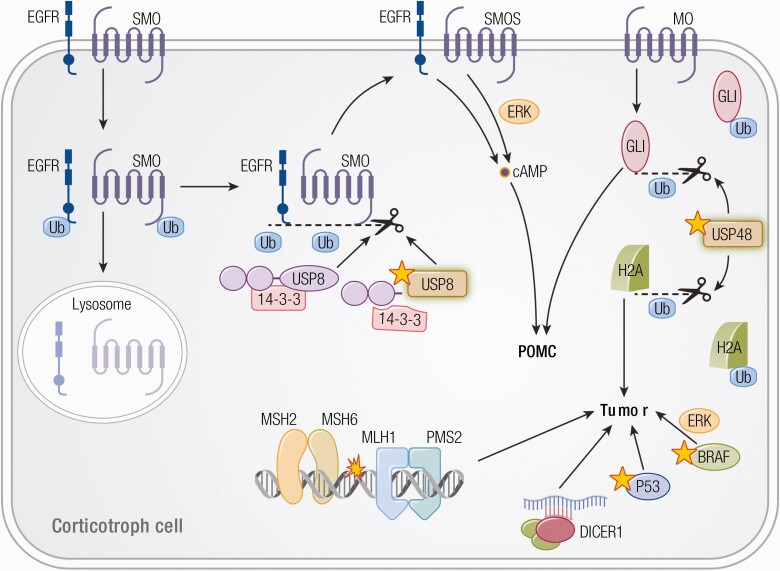
Tumorigenic mechanism in corticotroph cells (154). USP8 removes ubiquitin tags through its deubiquitinase action from its targets, such as EGFR and smoothened (SMO), preventing them from being degraded in the lysosome and allowing recycling back to the cell surface. Increased EGFR and SMO activity leads to increased cAMP signaling and POMC levels. Mutated *USP8* cannot bind 14-3-3 protein and undergoes cleavage, which increases its enzymatic activity, leading to increased deubiquitination of EGFR and SMO with higher expression on the cell membrane. Similarly, GLI1 and histone 2a (H2A) are suggested to be target of USP48 leading to increased activity with USP48 mutations. Loss-of-function of *DICER1, TP53, MLH1* and *MSH2* and gain-of-function of *BRAF* has also been suggested to be associated with corticotroph tumorigenesis.

Gain-of-function mutations in the deubiquitinase enzymes *USP8* and *USP48* are associated with ACTH-PTs (120, 132, 142, 154–161). *USP8* mutations disrupt the interaction between USP8 and the 14-3-3 protein, thereby allowing USP8 cleavage and increased enzymatic activity (154–156); this protects EGFR from lysosomal degradation, which leads to increased expression of EGFR (154–156) (Fig. 4) and pro-opiomelanocortin (*POMC)* (154, 159). Inhibition of USP8 leads to increased degradation of EGFR with suppresses corticotroph cell growth and ACTH secretion in vitro (162). Lapatinib, an EGFR inhibitor, decreases proliferation in vitro and reduces tumor weight in vivo (163). A recent meta-analysis showed an overall prevalence of 32% of *USP8* mutations in ACTH-PTs with a higher prevalence in females (164). *USP8*-mutated tumors are associated with an earlier onset (156, 165), smaller size (154), and increased ACTH production (154, 165). In patients who showed biochemical remission after surgery, the incidence of recurrence in a 10-year follow-up was higher in patients with *USP8* mutant tumors (165). In pediatric patients with Cushing’s disease, all recurrences after initial remission (in five patients) occurred in tumors with *USP8* mutations (166). When remission status was investigated, the remission rates were higher in patients with *USP8*-mutated-alleles, although no recurrence was detected for at least 6 months after surgery (164). These data suggest that patients with *USP8*-mutated-tumors may be more likely to go into initial remission post-surgery but may also more likely to show recurrence later in the clinical course. *USP8*-mutation-negative tumors are more likely to show sphenoid invasion with an increased epithelial-mesenchymal-transition signature (17). Recently, SSTR5 expression has been shown to be higher in *USP8*-mutated tumors (17, 159), potentially allowing the mutation status to be used as a predictor of response to pasireotide (a second generation SSA with greater affinity for SSTR5 (167)). In vitro studies found increased expression of pCREB and protein kinase A Cα on immunoblotting in AtT20 cells transfected with mutant USP8 (160).

Activating *USP48* mutations were found in 10% to 20% of ACTH-PTs (30, 132). *USP48* variants are associated with smaller tumors and better response to corticotropin releasing hormone (CRH) stimulation (132). Interestingly, both USP8 and USP48 have targets in the hedgehog signaling pathway: Smoothened for USP8 (168) and GLI1 for USP48 (132), suggesting that upregulation of this pathway may play a role in corticotroph mutagenesis (Fig. 4).

#### Novel Somatic Variants.

Analysis of whole exome and whole genome sequencing data from pituitary tumors has revealed a low number of somatic mutations per tumor across all subtypes (120, 142, 143). This is consistent with their generally low proliferation rate. Only a handful of genes show recurrent mutations (120, 141, 169). Such recurrently mutated genes are reported in more detail in Table 3. Identifying somatic variants in circulating free DNA has been attempted recently (170), and may develop into a useful method to follow patients with pituitary tumors.

**Table 3.** Novel recurrently mutated somatic variants identified through whole exome sequencing studies.

Gene Associated with Variant, Symbol	Gene Name	Hormone Subtype	Mechanism of Tumorigenesis	References
*NR3C1*	Nuclear receptor subfamily 3 group C member 1	ACTH-PT	Glucocorticoid receptor. If mutated, this receptor may become insensitive to feedback from cortisol leading to ACTH over-production (171).	(142, 154, 161, 172)
*MEN1*	Menin 1	Plurihormonal (GH/PRL)	Inactivating mutations underlie multiple endocrine neoplasia type 1, an autosomal dominant syndrome with pituitary tumors as part of the phenotype.	(118, 142)
*KIF5A*	Kinesin heavy chain isoform 5A	PRL, GT	Modulates cell proliferation. Somatic mutations also found in prostate cancer (173).	(142)
*GRB10*	Growth factor receptor bound protein 10	GH-PT	Suppresses signals from activated receptors tyrosine kinases, including insulin-like growth factor type 1 receptors. Inactivating mutations may allow increased signaling facilitating somatotroph tumorigenesis.	(142)
*BRAF*	BRAF proto-oncogene, serine/threonine kinase	ACTH-PT	Elevated kinase activity with activation of MAPK pathway and transactivation of POMC, which is the precursor of ACTH. Well-established oncogenic roles in melanoma and multiple carcinomas.	(160, 161)
*USP48*	Ubiquitin specific peptidase 48	ACTH-PT	USP48 has been suggested to increase transcriptional activation of POMC through the NF-κB pathway, increase response to CRH and possibly involve the hedgehog pathway.	(160, 161)
*PABPC1*	poly (A) binding protein cytoplasmic 1	ACTH-PT	Binds the poly (A) tail of mRNA and is involved in regulatory processes such as pre-mRNA splicing and regulation of nonsense-mediated decay.	(120)
*TP53*	Cellular tumor antigen p53	ACTH-PT	Well-established tumor suppressor with role in cell cycle arrest, DNA repair and apoptosis induction.	(132)
*SF3B1*	Splicing factor 3b subunit 1	PRL	–	(141)

Novel sequencing approaches have provided an opportunity to develop interesting insights into pituitary tumorigenesis:

1. **Novel candidate genes may point towards dysregulated pathways in tumorigenesis.** The first genome-wide association study of sporadic pituitary tumors identified new candidates (174): *CDK8* (cell cycle regulation) and *NEBL* and *PCDH15* (cell-cell adhesion). *CDK8* is an oncogene in colorectal (175) and gastric carcinoma (176) with differentially expressed genes from animal PRL-PT models showing enrichment for CDK8 targets (92). Targeted mutation profiling of canonical cancer-associated genes has identified recurrently mutated genes, with three main pathways implicated in tumorigenesis in one study: cell cycle regulation and growth, chromatin modification and transcriptional regulation, and DNA damage response (143).
2. **Subtype-specific mechanisms can be unveiled through WES and WGS studies focusing on tumor subgroups** (Table 4): WES and WGS studies on GH-PTs have identified dysregulation of multiple signaling pathways as potential tumorigenic mechanisms, although they need to be characterized further using functional studies. Mutations in different genes can affect the same pathways and mediate tumorigenesis. For example, several genes involved in cAMP signaling were found to have somatic variants in 14 of 36 somatotroph tumors, including G-protein-coupled receptors *CCR10* and *OR51B4*, which were suggested to increase cAMP signaling similar to classical GNAS mutations (43).

1. Pathways mediating aggressive behaviors such as local invasion (irrespective of subgroups) can be identified for further functional characterization: WES comparing invasive and noninvasive NFPTs and PRL-secreting tumors identified fifteen variants, which were mainly associated with processes involved in the regulation of invasion such as angiogenesis and cytoskeleton organization (179). ACTH-PTs with disrupted genomes and chromosomal instability are more likely to show cavernous sinus invasion (180).
2. Whole exome/genome sequencing studies can be used to identify copy number alterations and degree of chromosomal instability, which can provide further insights into subtype-specific tumorigenesis. Two distinct subgroups of pituitary tumors with low and high fractions of genomic disruption have been identified. The percentage of genome disruption used to define these 2 subgroups varies across studies (WES (17, 120, 142, 169, 180) and array-CGH (181)). Tumors with high genome disruption are enriched for hormone-secreting tumors, suggesting differing mechanisms of tumorigenesis between functioning and nonfunctioning tumors (17, 169). Chromosomal instability is not related to aggressiveness (17). GH-PTs show greater genomic disruption than ACTH-PTs as well as inactive tumors with no clinical evidence of hormone secretion (120). Subgroups of *GNAS*-mutation negative GH-secreting tumors show high levels of genomic instability (152) with 20 q amplification (*GNAS* locus) (17, 143, 181), which may be an alternative mechanism of increased signaling through *GNAS* driving somatotroph tumorigenesis. GH-PTs with recurrent aneuploidy showed high expression of pituitary tumor transforming gene 1 (PTTG1), which is a regulator of sister chromatid segregation, this may subsequently drive chromosomal instability (152, 182, 183).
3. Next generation sequencing approaches can also be used in sequencing extrachromosomal DNA and identifying their role in tumorigenesis: tumors with the highest number of mitochondrial variants show the highest Ki-67 indices, irrespective of tumor subtype (184), indicating a role for the mitochondrial genome in modulating tumor biology.

**Table 4.** Somatic variants identified from WES studies using specific tumor subtypes.

Tumor Subtype	Sequencing Technique	Insight into Tumorigenesis	References
GH-PT	WGS (42), WES (43)	No recurrent mutations; somatic variants mediating calcium signaling (42, 43), ATP signaling (42) and cAMP signaling (43) identified.	(42, 43)
ACTH-PT	WES	Enhanced promoter activity and increased transcription of POMC through different mechanisms can lead to tumorigenesis.	(161)
TSH-PT	WES	Six candidate variants identified, of which 2 have previously characterized tumorigenic roles: (a) Increased expression of SMOX is associated with gastric cancer, and (b) SYTL3 encodes proteins which interact with RAB27 and deregulation of this pathway is associated with bladder cancer.	(177)
NFPT	WES	Somatic variants in putative driver genes including platelet-derived growth factor D (PDGFD), N-myc down-regulated gene family member 4 (NDRG4), and Zipper sterile-motif kinase (ZAK) identified. However, these mutations were not replicated in the validation set.	(178)

## Epigenetic Mechanisms of Tumorigenesis

While germline and somatic genetic alterations have provided interesting insights into pituitary tumorigenesis, still the majority of pituitary tumors are sporadic with no known somatic driver mutations. Epigenetic changes are heritable phenotypic changes, which do not alter the DNA sequence. These can regulate transcription and/or translation. Epigenetic mechanisms may occur at the chromatin level, such as DNA methylation and histone modification, or at the RNA level, mainly mediated by noncoding RNAs, such as microRNAs, long noncoding RNAs (lncRNA), circular RNA (circRNA), and others. Such mechanisms affect gene expression and, consequently, tumorigenesis, and have assumed great significance, especially given the paucity of somatic mutations.

### DNA methylation

Recently, it has been shown that PIT-1 lineage tumors (GH-, PRL- and TSH-PTs) show global hypomethylation and cluster as a group with hypomethylation, chromosome alterations, and transposable element overexpression (17), suggesting that DNA hypomethylation may induce chromosomal instability through upregulation of transposable elements. *GNAS*-mutation positive GH-PTs also showed hypomethylation but with limited chromosomal alterations (17); *GNAS*-mutation positive tumors are associated with low levels of copy number alterations, as mentioned above.

Promoter methylation can prevent access to transcriptional machinery, leading to decreased expression. A systematic review of genes implicated in epigenetic dysfunction in pituitary tumors identified 16 tumor suppressor genes that underwent silencing secondary to methylation, of which 11 mediate apoptosis and cell cycle progression (185). These genes included *CDKN2A* and *RB1* (186, 187):

1. Methylation of CpG islands in *CDKN2A* is seen in up to 90% of sporadic pituitary tumors (185, 186, 188–190) with loss of expression of p16 on immunohistochemistry (188, 189).
2. Methylation of CpG islands in the *RB1* promoter in sporadic tumors (186, 190) is significantly associated with loss of expression on immunohistochemistry (191).
3. Transgenic mice with a disrupted *Rb1* allele develop pituitary tumors (192).

The systematic review also identified aberrant methylation in more than 50% of tumors in *GADD45γ*, *CASP-8, PTAG*, and *FGFR2* (185). *GADD45γ, CASP8*, and *PTAG* are involved in apoptosis modulation, while FGFR2 is a growth factor receptor. Growth factors and growth factors receptors can also be over-expressed in pituitary tumors (193, 194).

The epigenome may be modified by the de novo methyltransferases DNMT3A and DNMT3B. DNMT3A and DNMT3B is overexpressed in pituitary tumors (195, 196) and frequently detected in invasive tumors (195). Reducing the expression of DNMT3B was associated with increased levels of hypo-phosphorylated Rb, p21, and p27 proteins and reduced proliferation (196), suggesting that increased expression DNMT3B may mediate proliferation through promoter hypermethylation and silencing of tumor suppressor genes.

Gene imprinting is a process in which transcription of 1 allele is repressed through methylation, depending on the parent of origin. Relaxation of imprinting through loss of methylation can lead to increased transcription, such as in *GNAS*-mediated tumorigenesis (197). Imprinted genes such as *MEG3* (discussed later) have only 1 transcriptionally active copy, rendering them especially susceptible to inactivation through mutation or increased promoter methylation.

DNA methylation is associated with clinical characteristics, such as:

1. Tumor subtype: Microarray profiles differentiate between functioning tumors (including hormone subtypes) and NFPTs (120, 198–201) with GH-PTs divided into sparsely and 2 densely granulated groups (199).
2. Local invasion: While a study has shown distinct microarray profiles between invasive and noninvasive, nonfunctioning tumors (202), others have shown no significant differences, both when only NFPTs were used (203) or with a diverse group of functioning and nonfunctioning tumors (200). Interestingly, differentially methylated genes were enriched for cell adhesion pathways (202). Decreased LAMA2 (204) and WIF1 (205) expression (RT-qPCR and immunoblotting) with increased promoter methylation is seen in invasive NFPTs (204). LAMA2 overexpression in a xenograft model significantly suppressed tumor growth (204). Greater promoter methylation of *ESR1* and *RASSF1* is seen in noninvasive compared to invasive tumors (201).
3. Tumor size: *CDKN2A* methylation and reduced expression of p16 are significantly related to larger tumor size (188, 189). Macroadenomas show a greater frequency of promoter methylation of *MSH6* and *CADM1* than microadenomas (201).
4. Disease progression: *TERT* encodes a component of telomerase, which lengthens the telomere, mediates cell immortalization, and is a well-recognized oncogene. Aberrant *TERT* promoter methylation is associated with TERT upregulation in malignancies (206–208) and shorter progression-free survival and tumor recurrence (209) in pituitary tumors.
5. Implications for therapy: MGMT promoter methylation and consequent low expression are noted in a subset of pituitary tumors (210, 211). Temozolomide is currently used in the management of pituitary carcinomas and aggressive pituitary tumors (defined clinically as tumors that recur despite various treatment modalities such as surgery, radiotherapy, and pharmacological therapy (212–214)). Immunohistochemistry to assess MGMT expression has been recommended prior to commencing temozolomide, as high expression is associated with a lack of response, although the methodologies are not standardized and most clinicians would not use the result to discard temozolomide therapy (213, 214). Loss of MSH2 and MSH6 has also been linked to developing rapid resistance to temozolomide (215). Patients with somatotropinomas and *GSTP1* promoter methylation are more resistant to SSAs (216).

### Histone modification

Acetylation, methylation, and citrullination of histone tails are associated with active and inactive regions of the genome respectively. These epigenetic marks can be modified by chromatin regulators such as histone acetyltransferases, histone deacetylases, histone methyltransferases, and citrullination enzymes.

Several studies suggest that changes in histone acetylation play a crucial role in pituitary tumorigenesis:

1. Tumor-specific ikaros isoform Ik6 promotes pituitary cell survival through enhanced antiapoptotic activity by upregulation of Bcl-XL through promoter histone acetylation (217).
2. Expression of bone morphogenetic protein 4, which is a growth factor known to drive pituitary tumorigenesis (193), is controled through histone acetylation and methylation (218).
3. Tumor subtype-specific changes: Sirtuins are conserved histone deacetylases, which show differences in expression profiles in pituitary tumors based on size and hormone-secreting subtype (219). Histone deacetylase-2 deficiency is seen in glucocorticoid-resistant ACTH-PTs (220), with histone deacetylase-11 mediating decreased p53 expression in corticotroph AtT-20 cells (221). Inhibition of histone deacetylase activity reduces survival and ACTH secretion in corticotroph cells (222).
4. Aggressive behavior: Histone acetyltransferases p300 upregulates human pituitary tumor transforming gene (PTTG1) (223). PTTG1 is a growth factor with a well-established role in carcinogenesis and invasion, partly regulated through the c-myc pathway (224). A meta-analysis has confirmed increased PTTG1 expression in invasive pituitary tumors (225) (this has also been replicated in NFPTs (226)). Pituitary tumors show a global increase in H3K9 acetylation compared to normal pituitary, with increased acetylation seen in tumors with increased Ki-67 index (227).

Histone methylation and citrullination (conversion of arginine to citrulline catalyzed by peptidylarginine deiminase enzymes) may also mediate tumorigenesis. Enhanced H3K27 methylation and reduced H3K4/H3K9 methylation are found in tumors with increased RIZ1 expression (which is thought to be a histone methyltransferase), which also correlates with longer progression-free survival (228). Noninvasive tumors also show significantly increased RIZ1 expression compared to invasive tumors (228). Citrullination of histone H3 in GH3 cells represses the expression of specific tumor suppressor microRNAs, which leads to increased expression of known drivers such as N-MYC, and IGF-1 and increased proliferation (229).

### MicroRNAs

These are short noncoding RNAs that mediate post-transcriptional regulation of gene expression through RNA interference and mRNA destabilization. The role of microRNAs in pituitary tumorigenesis has been extensively reviewed elsewhere (230–232). The recent advances (mainly from the last 5 years) with novel insights into tumorigenesis are summarized in Table 5.

**Table 5.** Varying functions of microRNAs in pituitary tumorigenesis with illustrative examples from publications from the last 5 years.

Major Function	Mechanisms of Action and/or Relevant Examples	Supporting Evidence		
MicroRNAs can demonstrate a tumor suppressor action by targeting oncogenic gene products for degradation	MicroRNAs regulate the cell cycle, facilitating increased proliferation when deregulated (230).	miR-23b and miR-130b, targeting HMGA2 and cyclin A2 respectively, are downregulated in GH-PTs, GT-PTs and NFPTs (233). HMGA2 is a high mobility group protein, which shows increased expression in pituitary tumors (234, 235). HMGA2 overexpression enhances E2F1 activity and drives cell cycle (236, 237) microRNAs targeting HMGA2 and E2F1 are downregulated in pituitary tumors (235, 238).		
		miR-410 targeting the cyclin B1 gene is downregulated in GT-PTs (239).		
		miR-186 targets SKP2, which inhibits expression of p27, a negative regulator of G1 cell cycle progression, increasing proliferation. In human pituitary tumors, miR-186 and p27 expression is downregulated, while SKP2 expression is upregulated (240). In vitro, SKP2 overexpression decreases p27 expression and increases cell growth (240).		
	Multiple microRNAs, when down regulated, lead to increased expression of PTTG1 and its partners.	p53 activates transcription of miR-329, miR-300, miR-381, and miR-655 in pituitary tumor cells, which target PTTG1 (241).		
		miR-423-5p (targeting PTTG1) shows decreased expression in GH-PTs with increased PTTG1 expression compared to normal pituitary (242).		
		Overexpression of miR-524-5p downregulates expression of PTTG1 binding factor, which interacts with PTTG1 to mediate downstream effects (243) and significantly attenuates proliferation, migration, and invasion in folliculostellate cells (244); downregulation of this microRNA may mediate increased proliferation in the pituitary through PTTG1.		
	Other tumor-suppressive microRNAs which show reduced expression in human pituitary tumors or relevant cell lines.	miR-205-5p targeting CBX1 in pituitary cell lines (245).		
		miR‑1 targeting G6PD in human pituitary tumors (246).		
		miR-34a targeting SOX7 in GH4C1 cells (247).		
		miR-378 targeting RNF31 in human pituitary tumors (248).		
Increased expression of certain microRNAs can drive tumorigenesis by targeting gene products with tumor suppressor roles for degradation.	High levels of miR-107 (249) and miR-34 (250) target AIP mRNA in pituitary tumors.	miR-107 expression is significantly upregulated in GH-secreting and nonfunctioning pituitary tumors and inhibits in vitro AIP expression (249)		
		miR-34 is highly expressed in tumors with low AIP protein levels compared to tumors with high levels (250). miR-34 overexpression in HEK293 and GH3 cells inhibits endogenous *AIP* expression (250).		
MicroRNAs may regulate subtype-specific mechanisms of tumorigenesis.	Distinct profiles identified in tumor subtypes with differential microRNA expression specific to subtype.	Next generation sequencing and other techniques in GH-PTs, GT-PTs and NFPT subtypes (251, 252).		
		TSP-1, which has a tumor suppressor role, shows decreased expression in ACTH-PTs with increased miR-449c expression inhibiting its expression (253).		
		Four groups, miR1 to miR4, are strongly associated with tumor type with PIT1-lineage tumors being distinctly different from GT-PTs and ACTH-PTs (17).		
MicroRNAs play a prominent role in driving tumor invasion.	Decreased expression of mi-RNAs can have an anti-apoptotic effect, mediating invasion:	Downregulation of miR-132 and miR-15a/16 with upregulation of SOX5 is seen in invasive tumors (254). MiR-15a and miR-16-1 are also downregulated in pituitary tumors that develop after 12 months of age in mice with heterozygous *Men1* knockout (255). MiR-16 expression, which induces apoptosis (via Bax) and decreases proliferation, is reduced in pituitary tumors (256).		
		Invasive pituitary tumors show lower miR-21 expression with increased expression of its target, *PITX2*, which has an antiapoptotic role (257).		
		MiR-145-5p expression (targeting TPT1) correlates negatively with NFPT invasiveness. MiR-145-5p brings about apoptosis through Bcl-xL downregulation and Bax upregulation (258).		
		MiR-543 expression is increased in invasive tumors (259) and activates the Wnt/ β-catenin pathway by downregulating Smad7. Overexpression of miR-543 in HP75 cells increases cell proliferation, migration and invasion and decreases apoptosis (259).		
	microRNAs driving invasion specific to tumor subtype have also been identified:	MiR-183, which targets *KIAA0101* (a cell cycle activator), is downregulated in aggressive PRL-PTs and demonstrates an inverse correlation with Ki-67 indices (260).		
		MicroRNA 106b~25 cluster shows increased expression in invasive ACTH-PTs and Crooke cell adenomas (261). MiR-106b is upregulated in pituitary tumors and can increase migration and invasion of pituitary tumor cells through the phosphatidylinositol 3-kinase (PI3K)/AKT pathway (262, 263).		
		Differential microRNA profiles have been identified in invasive NFPTs (264).		
		MiR-26b (targeting *PTEN*) is upregulated and miR-128 (targeting *BMI1*) is down-regulated in GH-PTs compared to control and is shown to mediate growth and invasiveness of pituitary tumor cells (265). MiR-338-3p expression is increased in invasive GH-PTs and is mediated through upregulation of *PTTG1* (266).		
		The same microRNAs may even play different roles in different tumor subtypes: miR-410-3p significantly upregulates proliferation, invasiveness, cyclin B1 levels and activation of MAPK, PTEN/AKT, and STAT3 signaling pathways in gonadotroph and corticotroph cells but not in somatotroph cells (267).		
	Other microRNAs discovered recently through comparison of invasive and noninvasive pituitary tumors (target gene in parentheses):	Reduced expression of microRNA in invasive tumors:		
		microRNA	Targeted gene	Reference
		miR-145	*FSCN1*	(268)
		miR-124	*PTTG1IP*	(268)
		miR-183	*EZR*	(268)
		miR-148-3p and miR-152	*ALCAM*	(269)
		miR-200b	*PKCα*	(270)
		miRNA-145	*AKT3*	(271)
		Increased expression of microRNA in invasive tumors:		
		miR-26a	*PLAG1*	(272)
		miR-20a and miR-17-5p	*PTEN* and *TIMP2*	(273)

### Other noncoding RNAs

These include long noncoding RNA (lncRNA) (200 nt to ~100 kilobases long with no open reading frames (274)) and circular RNA (generated from exons of protein-coding genes and lacking a 5’ cap or 3’ poly (A) tail (275)). LncRNA and circRNAs have various functions, such as regulation of gene transcription and depletion of microRNA (“microRNA sponge”). Their up- or downregulation can result in tumorigenesis (275). Many lncRNAs are imprinted, allowing for dysregulation of imprinting resulting in abnormal function (274) (see above).

H19 expression is downregulated in pituitary tumors compared to normal pituitary (276) and H19 suppresses proliferation in vitro and in vivo (277) by inhibiting mTORC1 (276). CCAT2 is significantly upregulated in pituitary tumors with elevated expression correlating with poor prognosis (278). CCAT2 enhances proliferation in HP75 cells by suppressing PTTG1 degradation (278). IFNG-AS1 expression is greater in pituitary tumors with increased proliferation noted in HP75 cells on over-expression, probably mediated through ESRP2 (279). AFAP1-AS1 expression is also increased in pituitary tumors (280) and promotes proliferation by acting as a competing endogenous RNA of miR-103a-3p leading to activation of the PI3K/AKT pathway (281).

LncRNAs may drive subtype differentiation:

1. Genome-wide analysis of lncRNAs identified 839 differentially-expressed lncRNAs in GT-PTs compared to normal pituitary (282). Similar analysis in NFPTs identified 113 lncRNAs (283).
2. RPSAP52 expression is highly upregulated in GT-PTs and PRL-PTs, where it increases HMGA2 levels, compared with normal pituitary tissues (284). This relationship is not seen in GH-PTs.
3. LncRNA clarin 1 antisense RNA 1 (CLRN1-AS1) is downregulated in PRL-PTs therefore relieving inhibition from the Wnt/β-catenin pathway (285).
4. Overexpression of a subgroup of long noncoding RNAs, termed “highly up-regulated in liver cancer” (HULC), promoted GH3 cell viability, migration, invasion, and PRL and GH secretion with knockdown inducing apoptosis (286).

LncRNAs may also drive tumor invasion:

1. MEG3: This is an imprinted lncRNA, which is downregulated in NFPTs (287, 288). Loss of expression is not seen in other tumor subtypes (289, 290). Promoter hypermethylation mediates loss of expression (291). Ectopic expression inhibits growth in human cancer cell lines (287). MEG3 causes cell cycle arrest at the G1 phase with p53-dependent (292) and p53-independent (293) mechanisms. MEG3 expression is significantly reduced in invasive compared to noninvasive NFPTs (294).
2. HOTAIR: Expression is significantly higher in NFPTs compared to normal pituitary and in invasive compared to noninvasive NFPTs (294). HOTAIR interacts with the Polycomb Repressive Complex 2 (PRC2) (295, 296) and promotes invasion in pancreatic (297) and nonsmall cell lung cancer (298).
3. Lnc-SNHG1: Overexpression is seen in invasive pituitary tumors. Lnc-SNHG1 interacts with and decreases the activity of miR-302/372/373/520 in vitro, activating the TGFBR2/SMAD3 and RAB11A/Wnt/β-Catenin (299).
4. C5orf66-AS1: Its expression is decreased in invasive null cell adenomas compared to noninvasive tumors (300).
5. XIST: XIST and bFGF exhibited high expression while miR-424-5p showed low expression in invasive compared to noninvasive pituitary tumor tissue. XIST was found to up-regulate bFGF expression by competitively binding to miR-424-5p (301): bFGF (basic fibroblast growth factor) acts as a growth factor and can also promote angiogenesis.

Interestingly, multiple studies have shown a role for circRNAs in tumor progression in NFPTs. As discussed previously, miR-145-5p induces apoptosis in NFPTs; circOMA1 can promote tumor progression by sponging this microRNA (258). Differential circRNA expression profiles of invasive and noninvasive NFPTs have been shown (302, 303), with gene enrichment for cell adhesion and PI3K/AKT pathways (302). Ten circRNAs are upregulated in recurrent compared to primary tumors (302). In fact, a signature of two circRNAs (hsa_circ_0000066 and hsa_circ_0069707) is suggested to predict tumor recurrence in NFPTs (304).

### Global gene expression profiles

Novel sequencing and array-based approaches have allowed for:

1. **Identification of distinct profiles in subtypes.** Using RNA sequencing, 6 distinct transcriptomic profiles have been identified which match the World Health Organization (WHO) 2017 tumor classification (17), with the following discrepancies: (1) null cell subtype matches GT-PTs; (2) silent and secreting ACTH-PTs show distinct profiles; (3) mixed GH-PRL tumors cluster with GH-PTs, rather than PRL-PTs; and (4) sparsely granulated GH-PTs cluster with thyrotroph and plurihormonal PIT1-positive tumors rather than densely granulated GH-PTs. Distinct tumor type-specific profiles were shown in earlier studies as well: GT-PTs (282, 305-307), ACTH-PTs (120, 308-310), GH-PTs (120, 311), PRL-PTs (312, 313) and NFPTs (120, 314, 315). Insights from such studies include a confirmation of the importance of deregulation of the cell cycle in pituitary tumorigenesis across subtypes (17, 306, 307, 311, 314, 316), deregulation of the mTOR signaling pathway in GT-PTs (282), and alterations in the Notch pathways in PRL-PTs (312) and NFPTs (315). A subtype of NFPTs may actively suppress the immune system, raising the possibility of immunotherapy in treatment (317). Integration of microarray datasets has also been used to identify immune-related genes and further explore this possibility (318). Recently, evaluation of splicing machinery components in GH-PTs, ACTH-PTs, and PRL-PTs showed severe dysregulation in all subtypes compared to normal pituitary (319).
2. In a subgroup of tumors from patients with acromegaly, ectopic GIPR overexpression was associated with a paradoxical increase in GH after an oral glucose tolerance test (70, 71). These GIPR-expressing somatotropinomas are negative for *GNAS* mutations (70, 72). An overall hypermethylator phenotype was identified in GIPR-expressing samples (compared with *GNAS*-mutated tumors), which also showed hypermethylation in the GIPR gene body, potentially driving ectopic expression (70). This represents a novel tumorigenic mechanism, similar to the well-described ectopic receptor induced adrenal tumors.
3. A study on invasive pituitary tumors identified that the TNFα network—including genes coding for proteins (TNFα, CCL3, CXCL12, and CCL2), microRNAs (miR-181c-5p and miR-454-3p) and lncRNAs (NR_033258 and lncRNA_SNHG24)—is upregulated in bone-invasive pituitary adenomas compared nonbone-invasive counterparts, suggesting that targeting the TNFα pathway may be beneficial for these invasive tumors (320).

### Proteomics

Novel techniques such as nanoscale liquid chromatography coupled to tandem mass spectrometry (nano LC-MS/MS) have been used recently (321) in determining proteomic profiles. Integration of such techniques with transcriptomics has allowed for identification of invasion-related biomarkers (322) and pathways (323) in NFPTs. This technique has even allowed for the identification of novel pattern of phosphorylation in GH-PTs (321), with enrichment of differentially phosphorylated proteins in glycolysis and AMPK signaling. High-performance liquid chromatography coupled to mass spectrometry has been used to identify differentially-expressed molecules in fibroblasts isolated from bone-destructive NFPTs (324) (with significant upregulation of osteopontin, which can stimulate cell migration and invasion (325)). The first study to investigate protein ubiquitination profiling in pituitary tumors compared to normal pituitary showed enrichment for the PI3K/AKT signaling pathway in NFPTs (326).

High-resolution Fourier transform mass spectrometry is another promising novel technique which has identified 105 novel proteins in the normal anterior pituitary compared with previous high-throughput proteomic-based studies (327). Using this approach may also identify new candidate proteins and/or pathways driving pituitary tumorigenesis. Previously used techniques in proteome profiling of pituitary tumors include two-dimensional gel electrophoresis-based comparative proteomics (312, 315, 328, 329) and protein immunoblot array analysis (330).

## Other Factors Influencing Pituitary Tumorigenesis

In this review we have concentrated on the novel genetic changes in pituitary adenomas. We refer to data on other important aspects of pituitary tumorigenesis, such as the role of senescence (331, 332), cytokines (47, 333) or tumor-associated fibroblasts (334, 335) in establishing the clinical phenotype of tumors is an emerging focus of research interest (336).

## Conclusions

Novel mechanisms of pituitary tumorigenesis have been identified in recent years, both pertaining to germline mutations underlying familial tumors and somatic mutations and epigenetic changes driving sporadic tumors. Epigenetic modifications have become increasingly important in understanding tumorigenesis, as most pituitary tumors are sporadic with no known genetic driver mutations (with the exception of a significant proportion of GH-PTs and ACTH-PTs with *GNAS* and *USP8* mutations, respectively). While novel somatic variants have been identified through whole genome and exome sequencing, their role in driving sporadic tumors remains to be established through further functional studies. Epigenetic changes at the chromatin (pretranscription) and RNA levels (post-transcription) are especially crucial in determining clinical characteristics such as subtype differentiation and local invasion (occasionally through epigenetic mechanisms specific to subtype). Indeed, a novel recent multiomic classification system using somatic mutations, chromosomal alterations and the miRNome, methylome, and transcriptome has shown the PIT1 lineage to be the main separator driving distinct group classification (17). Such integrated approaches to these genetic and epigenetic mechanisms will permit identification of molecular mechanisms of tumorigenesis common across different subtypes, as well as specific to tumor subtype, allowing for the development of novel therapeutic strategies.

## Data Availability

Data sharing is not applicable to this article as no datasets were generated or analyzed during the current study.

## Abbreviations

ACTH-PT: adrenocorticotropic hormone-secreting pituitary tumor
circRNA: circular RNA
FIPA: familial isolated pituitary adenomas
GH-PT: growth hormone-secreting pituitary tumor
GT-PT: gonadotropin (FSH/LH)- secreting pituitary tumor
Gαi: inhibitory Gα protein subunit
HGVS: Human Genome Variation Society
lncRNA: long noncoding RNAs
PitNET: pituitary neuroendocrine tumor
PRL-PT: prolactin-secreting pituitary tumor
PT: pituitary tumor
SSA: somatostatin analogue
TSH-PT: thyroid stimulating hormone-secreting pituitary tumor
WES: whole exome sequencing
WGS: whole genome sequencing
XLAG: X-linked acrogigantism

## Additional Information

***Disclosure Summary:*** The authors have nothing to disclose.
